# Acacetin exerts antitumor effects on gastric cancer by targeting EGFR

**DOI:** 10.3389/fphar.2023.1121643

**Published:** 2023-05-17

**Authors:** Guangtao Zhang, Jiahuan Dong, Lu Lu, Yujing Liu, Dan Hu, Yuanmin Wu, Aiguang Zhao, Hanchen Xu

**Affiliations:** ^1^ Longhua Hospital, Institute of Digestive Diseases, Shanghai University of Traditional Chinese Medicine, Shanghai, China; ^2^ Shanghai Frontier Research Center of Disease and Syndrome Biology of Inflammatory Cancer Transformation;, Shanghai, China; ^3^ Department of Oncology, Longhua Hospital, Shanghai University of Traditional Chinese Medicine, Shanghai, China; ^4^ Shanghai Pudong New Area Hospital of Traditional Chinese Medicine, Shanghai, China

**Keywords:** gastric cancer, acacetin, cell apoptosis, target, EGFR

## Abstract

**Background:** Gastric cancer (GC) is a common malignant tumor with a poor prognosis. Combination treatments may prolong the survival of patients with GC. Acacetin, which is a flavonoid, exerts potent inhibitory effects on several types of cancer cells; however, the mechanisms of action remain poorly understood.

**Methods:** Network pharmacology and RNA sequencing were used to predict the targets of acacetin, which were then verified by drug affinity responsive target stability (DARTS), cellular thermal shift assay (CETSA) and molecular docking. The biological functions of acacetin in MKN45 and MGC803 cells were investigated using TUNEL assays, crystal staining and colony formation assays. The pathways affected by acacetin were verified through reverse experiments. The *in vivo* antitumor efficacy of acacetin was assessed in a subcutaneous xenotransplanted tumor model.

**Results:** In this study, we identified EGFR from more than a dozen predicted targets as a protein that directly binds to acacetin. Moreover, acacetin affected the level of phosphorylated EGFR. *In vitro*, acacetin promoted the apoptosis of GC cells. Importantly, EGFR agonists reversed the inhibitory effects of acacetin on the STAT3 and ERK pathways. *In vivo*, acacetin decreased the protein levels of pEGFR in tumors, resulting in increased GC xenograft tumor regression without obvious toxicity.

**Conclusion:** Our findings highlight EGFR as one of the direct targets of acacetin in GC cells. Acacetin inhibited the phosphatase activity of EGFR *in vitro* and *in vivo*, which played a role in the antitumor effects of acacetin. These studies provide new evidence for the use of acacetin as a potential reagent for the treatment of GC.

## Introduction

Gastric cancer is the fifth most common cancer and the third leading cause of cancer-related death worldwide (Smyth et al., 2020); nearly half of all gastric cancer cases occur in China (Wang et al., 2021). The main treatment for gastric cancer is surgical resection followed by radiotherapy or chemotherapy. Advances have been made in targeted therapy and immunotherapy, and many patients have already benefited from these therapeutic approaches. However, heterogeneous efficacy is still observed in the treatment of gastric cancer, and this heterogeneity can lead to poor prognosis and survival. Hence, there is an urgent clinical need to seek new molecular targets and develop drugs for the treatment of this disease.

Acacetin is a natural flavone with therapeutic potential for many diseases, and it is widely found in plants and dietary sources. Various biological antioxidant and anti-inflammatory activities of acacetin, especially anticancer activities, have been reported, and subsequent research has already advanced the understanding of these activities (Singh et al., 2020). In addition to promoting apoptosis (Alfwuaires et al., 2022), acacetin decreases the expression of VEGF to suppress tumor angiogenesis (Bhat et al., 2013). Moreover, acacetin can also inhibit the invasion and migration of prostate and NSCLC cells by regulating p38 MAPK signaling (Chien et al., 2011). Although the effects of acacetin have been validated, the targets of acacetin are not completely clear. One study found that STAT3 is a direct target that is bound by acacetin in DU145 prostate cancer cells (Yun et al., 2021). In our previous study (Zhang et al., 2022), we found that acacetin not only suppressed cell viability in a dose- and time-dependent manner but also influenced epithelial-mesenchymal transition (EMT) in GC cells. However, since acacetin is a natural product with multiple targets, other targets of acacetin are still being explored. In this study, the process of exploring and validating targets of acacetin was elucidated. Meanwhile based on the function of the targets, downstream of the signals have been further identified and reversed. Therefore, acacetin is a therapeutic drug that shows promise for clinical translation and development for the treatment of gastric cancer.

## Materials and methods

### Reagents

Acacetin (purity 98%) was purchased from Tauto Biotech Co., Ltd. (Shanghai, China) and dissolved in DMSO. Primary antibodies against PI3K p85 (4292), EGFR (4267), Met (8198), GSK3β (9315), MMP9 (13,667), p-EGFR (4404 and 2234), Bax (2774), Bcl-2 (17,447), Bcl-xl (2764), Cleaved Caspase-3 (9661), Caspase-3 (9662), Cleaved PARP (5625), PARP (9532), p-STAT3 (9145), STAT3 (9139), p-ERK (4370), Erk1/2 (4695), PCNA (13,110), and Ki-67 (9027), as well as HRP-linked anti-rabbit IgG (7074) and HRP-linked anti-mouse IgG (7076) were purchased from Cell Signaling Technology (Danvers, MA, United States). Antibodies against PTGS2 (D223097), HAVCR1 (D260086), TMPRSS4 (D123375), GJB4 (D260904), KRT17 (D120232), EGR1 (D120585) and AKR1B10 (D221558) were obtained from Sangon Biotech (Shanghai, China). An antibody against GAPDH (60,004-1) was purchased from Proteintech (Wuhan, China). Human EGF (AF-100-15) was purchased from Peprotech (United States).

### Cell lines

Two GC cell lines, namely, MKN45 cells (gastric signet-ring cell carcinoma cell line that originated from lymph node metastasis) and MGC803 cells (poorly differentiated mucinous adenocarcinoma cell line), were purchased from the Shanghai Institute of Cell Biology, Chinese Academy of Sciences (Shanghai, China). Both cell lines were grown in RPMI-1640 medium (Gibco) supplemented with 10% FBS (Gibco).

### Network pharmacology

The targets of acacetin were identified by Swiss target prediction (http://www.swisstargetprediction.ch/) (Daina et al., 2019) and Traditional Chinese Medicine System Pharmacology (TCMSP, http://lsp.nwu.edu.cn/browse.php) (Ru et al., 2014). GC-related genes were screened using the GeneCards database (https://www.genecards.org/) (Luo et al., 2021). Then, by searching the UniProtKB database (https://www.uniprot.org/) or STRING database (https://string-db.org/), the target proteins were converted to gene symbols. The STRING database was used to construct a PPI (protein-protein network). The compound-target-pathway network analyses were performed using Cytoscape 3.7.2 software. Gene Ontology (GO) and Kyoto Encyclopedia of Genes and Genomes (KEGG) pathway analyses were performed with the DAVID 6.8 database (https://david.ncifcrf.gov/).

### RNA sequencing

MKN45 cells were treated with solvent control (DMSO) or acacetin (40 μM) in three biological replicates for 24 h. TRIzol reagent was added to extract total cellular RNA. Total RNA was analyzed by a NanoDrop ND-2000 spectrophotometer and Agilent Bioanalyzer 4200 (Agilent Technologies, Santa Clara, CA, United States) for quality assessment, and qualified RNA was used for subsequent library construction. The constructed libraries were analyzed to determine their concentrations and sizes. Illumina sequencing was performed on the qualified libraries to obtain the sequence information of the tested fragments. Data analysis was carried out according to established processes. The sequencing and analysis in this section were completed by Shanghai Outdo Biotech Co., Ltd.

### Drug affinity responsive target stability

After MKN45 and MGC803 cells had reached a certain confluence, the supernatants were removed, and the cells were washed 3 times with PBS. An appropriate amount of NP-40 lysis buffer was added, and the cells were incubated on ice for 30 min. The cell lysis products were collected and centrifuged at 4°C for 10 min at 12,000 rpm. The supernatants were transferred to new precooled EP tubes, and the sample concentrations were measured by BCA assay. Different concentrations of acacetin (100 μM and 500 μM) were added to the supernatants and incubated at room temperature for 1 h. Pronase (original concentration of 10 mg/mL) was diluted in 1× TNC solution, and the mixture was added in different ratios according to protein concentration (total protein: Pronase ratios of 10,000:1, 8000:1, 6000:1). The samples were incubated at room temperature for 30 min. The reactions were terminated by high-temperature denaturation with the addition of 5× loading buffer, and Western blotting was performed. The experiment was repeated ≥3 times.

### Cellular thermal shift assay

Cell lysates were collected and incubated with acacetin or DMSO for 1 h at room temperature. The lysate of each sample was heated for 3 min at the indicated temperature of 55°C–75°C, and then, the samples were cooled for 3 min at room temperature. The samples were centrifuged for 10 min at 12,000 rpm, and then, the supernatants were analyzed by Western blotting.

### Molecular docking

The structure of the small molecule compound acacetin that was used in this docking experiment was obtained from the PubChem database, and the energy of this compound was minimized and saved by the MM2 module in Chem3D software. The crystal structure of the target protein EGFR was downloaded from the protein data bank (https://www.rcsb.org/). The processing and optimization of virtual screening were performed using Schrödinger Maestro software. The docking results were visualized by PyMOL 2.1 software. The modes of compound and protein interactions were analyzed to identify the interaction with each target protein residue, and then, the docking of the compounds was scored to determine whether the screened compounds had a certain active effect.

### TUNEL assay

Adherent cells and acacetin-treated cells were fixed in a 24-well plate, permeabilized with 0.3% Triton X-100 PBS, and incubated with the TUNEL mixture from the One Step TUNEL Apoptosis Assay Kit (Beyotime Biotechnology, China) according to the manufacturer’s instructions. The stained wells were washed with PBS three times, stained with DAPI and measured by ImageXpress Mico4 (Molecular Devices, United States).

### Crystal staining

For crystal staining, 1 × 10^4^ cells were seeded in each well of a six-well plate and incubated until the cells adhered. The adherent cells were exposed to acacetin for 24 h and then fixed in a 6-well plate with methanol (China-reagent, China) for 1 h. Then, the cells were stained with 1% crystal violet stain solution (Solarbio, China) for another 1 h, washed with water and photographed.

### Colony formation assays

Approximately 500 GC cells/well were cultured in a 6-well plate with DMEM and exposed to acacetin for 2 days. After the medium was changed to fresh medium, the cells were allowed to grow for another 10 days until colonies were formed. Then, the cells were fixed and stained with crystal violet stain solution as described above.

### Real-time PCR analysis

Reverse transcription of RNA to cDNA using a reverse transcription assay kit (Life Technologies, Carlsbad, CA, United States). mRNA expression was determined using the SYBR Green PCR Mix (Thermo Fisher Scientific, Waltham, MA, United States). The relative expression of each gene was calculated using the 2^−ΔΔCT^ method. The expression was normalised to the average expression of all control individuals. The housekeeping gene used was β-actin. The following primer pairs were used: EGFR, 5′-AGG​CAC​GAG​TAA​CAA​GCT​CAC-3’ (forward)and 5′-ATG​AGG​ACA​TAA​CCA​GCC​ACC-3’ (reverse); and β-actin, 5′-CCC​ATC​TAT​GAG​GGT​TAC​GC-3’ (forward) and 5′-TTT​AAT​GTC​ACG​CAC​GAT​TTC-3’ (reverse).

### Protein extraction from cells and tissues and Western blotting analysis

To extract proteins from cells, RIPA buffer (supplemented with a protease inhibitor and phosphatase inhibitor) was added to the cells. Then, the cells were incubated on ice for 30 min, sonicated for 15 s, and centrifuged at 12,000 rpm at 4°C for 10 min. The supernatants were then used for Western blotting. Tumor tissues were dissected on ice and homogenized with a tissue grinder, and then, the samples were sonicated and collected for Western blotting. In this experiment, the protein concentrations of all the samples were measured to normalize the amount of protein that was used for each sample. Different protein concentrations or cell numbers per well were tested to ensure that the signals were in the linear range. Background corrections were performed by subtracting the background signals from the blank samples. The amount of protein that was loaded in each lane was 15-30 μg. Western blotting was performed in a manner that was consistent with our previously described protocols.

### GC xenograft models

All the animal studies were performed according to the Guide for the Care and Use of Laboratory Animals approved by Longhua Hospital, Shanghai University of TCM. Male BALB/c-nude mice (4–6 weeks old) were obtained from GemPharmatech Co., Ltd. (Nanjing, China) and housed under specific pathogen-free (SPF) conditions. For the MKN45 xenograft experiment, xenografts were established by subcutaneous injections of 2 × 10^6^ MKN45 cells. When the tumors reached 50-100 mm^3^, the mice were randomly divided into the control group and acacetin treatment groups (25 mg/kg and 50 mg/kg) (n = 5). Acacetin was administered every 2 days for 3 weeks via intraperitoneal injection. Tumor volume was calculated using the formula: volume (mm^3^) = length × width × width/2. The mice were euthanized, and the tumors, serum and major organs, such as the liver, kidney, and lung, were isolated.

### Tissue staining

Tissue samples were washed with PBS to remove the blood, and large tissues were cut open and fixed with 4% paraformaldehyde. Tissue samples of appropriate size were placed in an embedding box. The tissues were dehydrated and embedded in paraffin. HE and immunohistochemical staining were performed according to the manufacturer’s instructions. Images were captured under a microscope.

### Safety evaluation

MKN45 tumor-bearing mice were treated as described above. On Day 21, blood was collected, allowed to clot and centrifuged to obtain serum samples for the ALT and AST tests. Enzyme-linked immunosorbent assay (ELISA) was performed according to the manufacturer’s instructions.

### Statistical analysis

All the experiments were performed at least three times in duplicate. Student’s t-test was used to analyze differences between the two groups, and these analyses were performed with SPSS 23.0 (SPSS, United States) and GraphPad Prism 8 (GraphPad, United States) software. For the tests, ^*^ *p* < 0.05, ^**^ *p* < 0.01 and ^***^ *p* < 0.001 indicate statistically significant differences compared with the control group; similarly, ^#^ *p* < 0.05, ^##^ *p* < 0.01 and ^###^ *p* < 0.001 indicate statistically significant differences compared with the EGF-treated group.

## Results

### Identification of common targets of GC and acacetin through network pharmacology

**FIGURE 1:**
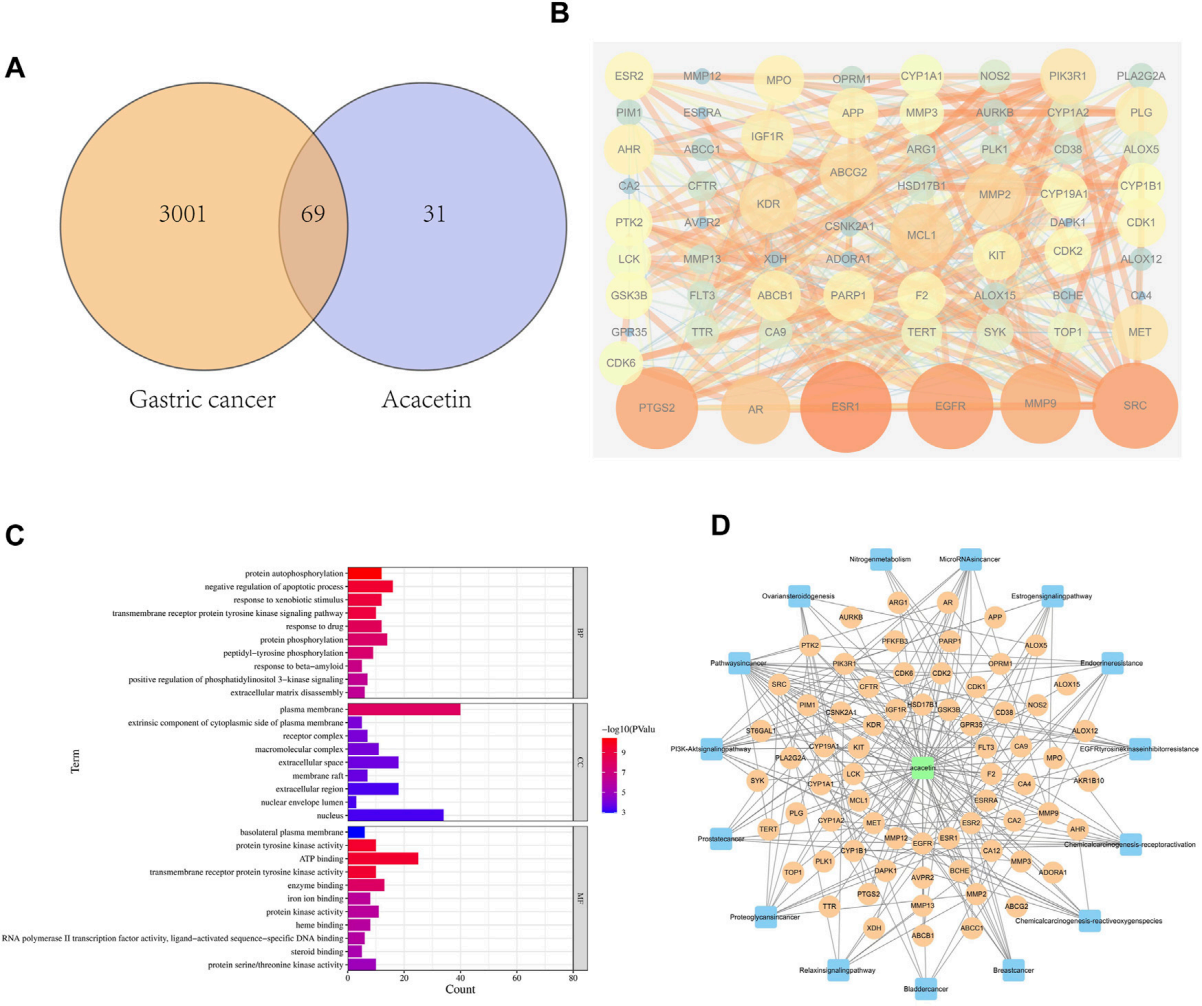
Network pharmacology predicted the target of acacetin in GC. **(A)** The Venn diagram showing the target genes through which acacetin affects GC. **(B)** PPI network between acacetin and GC. **(C)** Analysis of the biological functions of the targets and their related pathways. **(D)** Component-target-pathway network showing the potential pathways through which acacetin functions in the treatment of GC.

To predict potential common targets between GC and acacetin, 3070 GC-related targets were identified from the GeneCards database. In addition, the chemical structure of acacetin was obtained from PubChem, and 100 related targets were predicted by Swiss target prediction. Sixty-nine potential common gene targets were identified by filtering the overlapping targets of GC and acacetin (Figure 1A). A protein‒protein interaction (PPI) network was established to visualize the relationships among the identified common targets (Figure 1B). There were 370 lines and 65 nodes in the PPI network. We performed GO analysis through the Metascape platform, and the most common targets were related to protein kinase activity (Figure 1C). The network of common targets and signaling pathways was constructed using Cytoscape (Figure 1D). We imported the PPI network into Cytoscape analysis software, and the top 10 genes were screened and verified by subsequent experiments according to the closeness centrality and degree. These targets were predicted to be the potential targets of acacetin in the treatment of GC.

### Transcriptome of GC cells treated with acacetin

**FIGURE 2:**
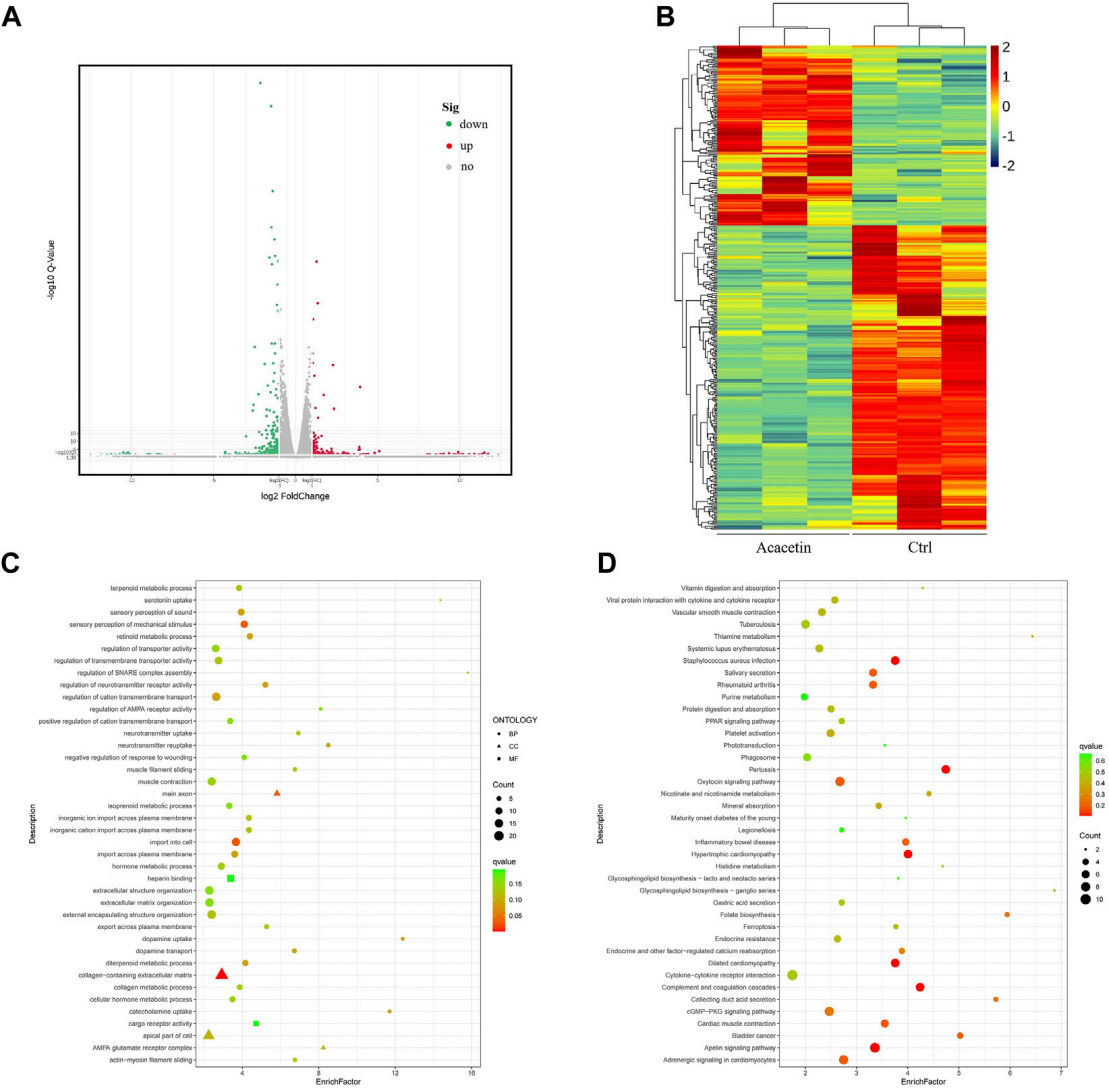
Transcriptome sequencing was performed in MKN45 cells after treatment with acacetin. **(A)** Analysis of differentially expressed genes. **(B)** Visual clustering heatmap of the FPKM values that corresponded to the differentially expressed mRNAs. **(C, D)** GO and KEGG functional enrichment analyses of differentially expressed genes.

To determine the changes in gene expression induced by acacetin, RNA-seq was used to analyze the RNA expression patterns of MKN45 GC cells after acacetin treatment. Between the control DMSO group and the acacetin group, 315 genes were differentially expressed (*p* < 0.05, log(fold change) > 2). Among these genes, 261 genes were downregulated and 154 genes were upregulated in the acacetin group (Figure 2A). Visualization of the FPKM values that corresponded to the identified differentially expressed mRNAs by clustering heatmap revealed that the two groups possessed distinct hierarchical gene clustering (Figure 2B). All the differentially expressed genes were enriched by Gene Ontology (GO) and Kyoto Encyclopedia of Genes and Genomes (KEGG) analyses to further explore the molecular biological functions and related pathways in acacetin-treated GC cells. Among them, genes related to tumor apoptosis, invasion and metastasis signaling pathways were significantly enriched (Figures 2C,D). In this part, genes with greater than the 4-fold differential expression that was related to tumors were selected for subsequent target verification.

### EGFR is one of the direct targets of acacetin

**FIGURE 3:**
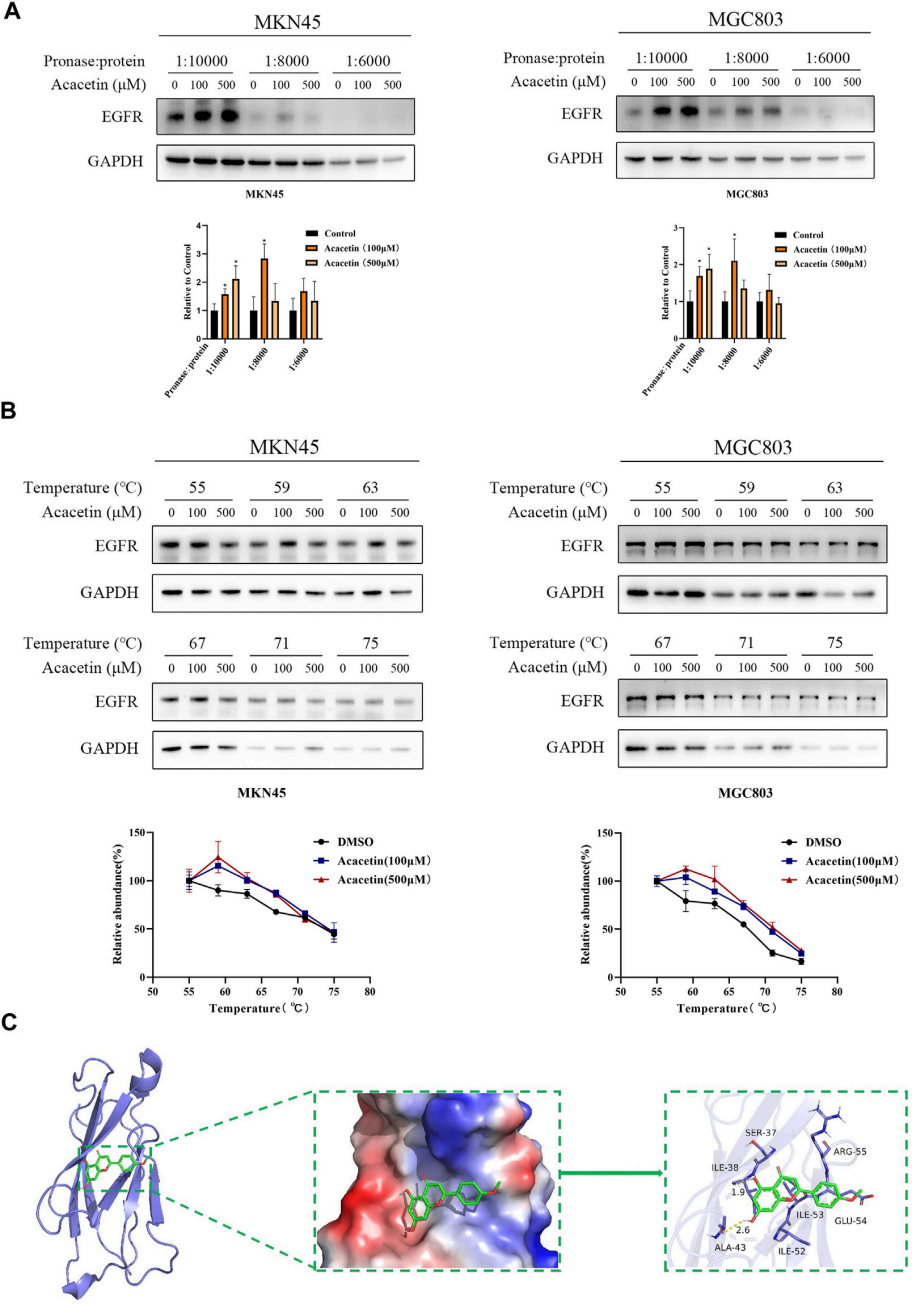
Acacetin directly interacted with EGFR. **(A)** MKN45 and MGC803 cell lysates were incubated with acacetin (100 μM and 500 μM) or DMSO and then analyzed by Western blotting. **(B)** Acacetin increased the thermal stability of EGFR compared with the control at a series of temperatures from 55°C to 75°C. **(C)** The predicted model of the interaction between acacetin and EGFR. The data are shown as the mean ± SD. *p < 0.05 *versus* control.

The specific targets of acacetin in GC remain unclear. Therefore, potential targets were predicted by network pharmacology and RNA-seq in this study. The acacetin and GC targets that were selected for verification are as follows: PIK3R1, EGFR, Met, PTGS2, GSK3β, and MMP9 according to network pharmacology and HAVCR1, TMPRSS4, GJB4, KRT17, EGR1, and AKR1B10 according to RNA sequencing. Then, multiple drug-protein binding experiments were carried out. The DARTS assay showed that when the cells were treated with different concentrations of pronase, acacetin partially inhibited the effects of pronase on digesting EGFR (Figure 3A) and Met (Supplementary Figure S1) compared with the control in a dose-dependent manner. In contrast, the remaining potential targets did not exhibit this effect (Supplementary Figure S1). The DARTS assay was performed simultaneously in MKN45 and MGC803 GC cells. Next, acacetin treatment had little effect on Met (Supplementary Figure S2), so EGFR was selected for further verification. The CETSA assay was performed with lysates of the 2 cell lines. The results showed that under different temperatures, EGFR accumulation increased after acacetin treatment compared with DMSO treatment (Figure 3B). In addition, molecular docking data indicated that acacetin had a strong affinity for EGFR (Figure 3C). Taken together, these results confirmed that acacetin is directly bound to EGFR.

### Acacetin suppresses the tyrosine phosphorylation of EGFR and leads to GC cell apoptosis

**FIGURE 4:**
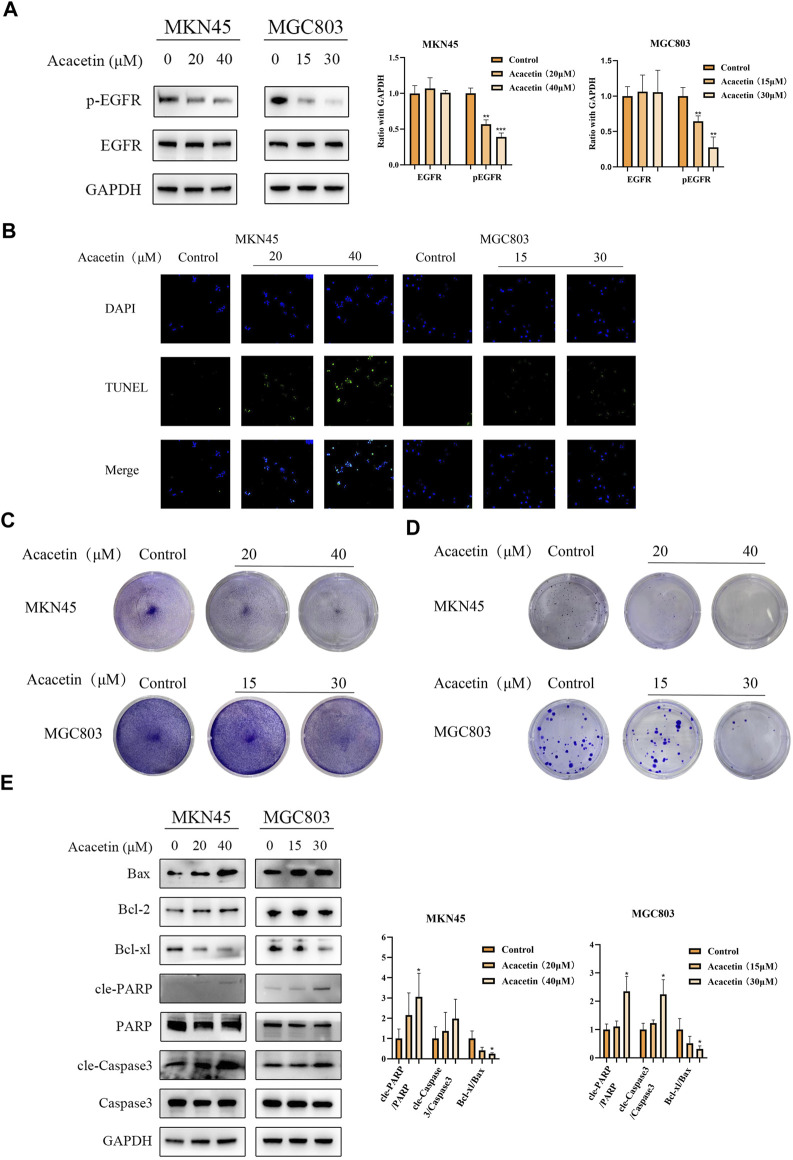
Acacetin suppressed the tyrosine phosphorylation of EGFR and led to GC cell apoptosis. **(A)** Acacetin inhibits the phosphorylation of EGFR at residue 1148. **(B)** TUNEL assays of GC cells that were stained with DAPI (blue) or fluorescein-dUTP (green) (magnification, ×40). **(C)** GC cells were treated with different concentrations of acacetin and **(C)** subjected to crystal staining or **(D)** analyzed by clonogenic assay. **(E)** After treatment with acacetin, the expression levels of apoptosis-related proteins were analyzed by Western blotting. The data are shown as the mean ± SD. *p < 0.05, ^**^ *p* < 0.01 and ^***^ *p* < 0.001 *versus* control.

To determine the effect of acacetin on the mRNA expression of EGFR, we performed real-time PCR (RT-PCR). The expression of EGFR was not significantly changed in MKN45 cells after acacetin intervention, while MGC803 cells were increased in the high-dose group (Supplementary Figure S3). Then, it was necessary to further study the inhibitory effect at the protein level. The inhibitory concentration of acacetin in MKN45 and MGC803 cells was determined according to our previous data. GC cells were exposed to different concentrations of acacetin for 24 h, and there was no difference in protein expression according to Western blotting (Figure 4A). Therefore, we examined the effects of acacetin on EGFR phosphorylation. Several phosphorylated residues were detected (including residues 1068 and 1148); surprisingly, the phosphorylation of residue 1148 of EGFR could be significantly inhibited by acacetin (Figure 4A and Supplementary Figure S4). Activated EGFR performs a variety of biological functions, such as functions related to tumor growth, survival, and progression (Wong, 2011). Therefore, we examined the effects of acacetin on various malignant phenotypes of GC cells. Apoptosis plays a pivotal role in the pathogenesis of the disease. In this study, acacetin significantly enhanced apoptosis as determined by TUNEL assays (Figure 4B). It was also confirmed by crystal staining that acacetin suppressed the growth of MKN45 and MGC803 cells (Figure 4C). Moreover, acacetin significantly decreased the colony formation of MKN45 and MGC803 cells, which suggested long-term inhibitory effects (Figure 4D). The underlying mechanism is related to the Bcl family, caspase family and PARP protein (Carneiro and El-Deiry, 2020). The expression of these proteins was explored, and acacetin not only decreased the Bcl-xl/Bax ratio but also increased PARP expression, which demonstrated that acacetin induced apoptotic signaling. Although there was no significance in Caspase3 activation in MKN45 cells, there was a clear trend of upregulated cleaved-Caspase3 expression (Figure 4E). Hence, acacetin could inhibit growth and promote apoptosis via Caspase and PARP signaling.

### Acacetin plays an anticancer role by regulating pathways downstream of EGFR

**FIGURE 5:**
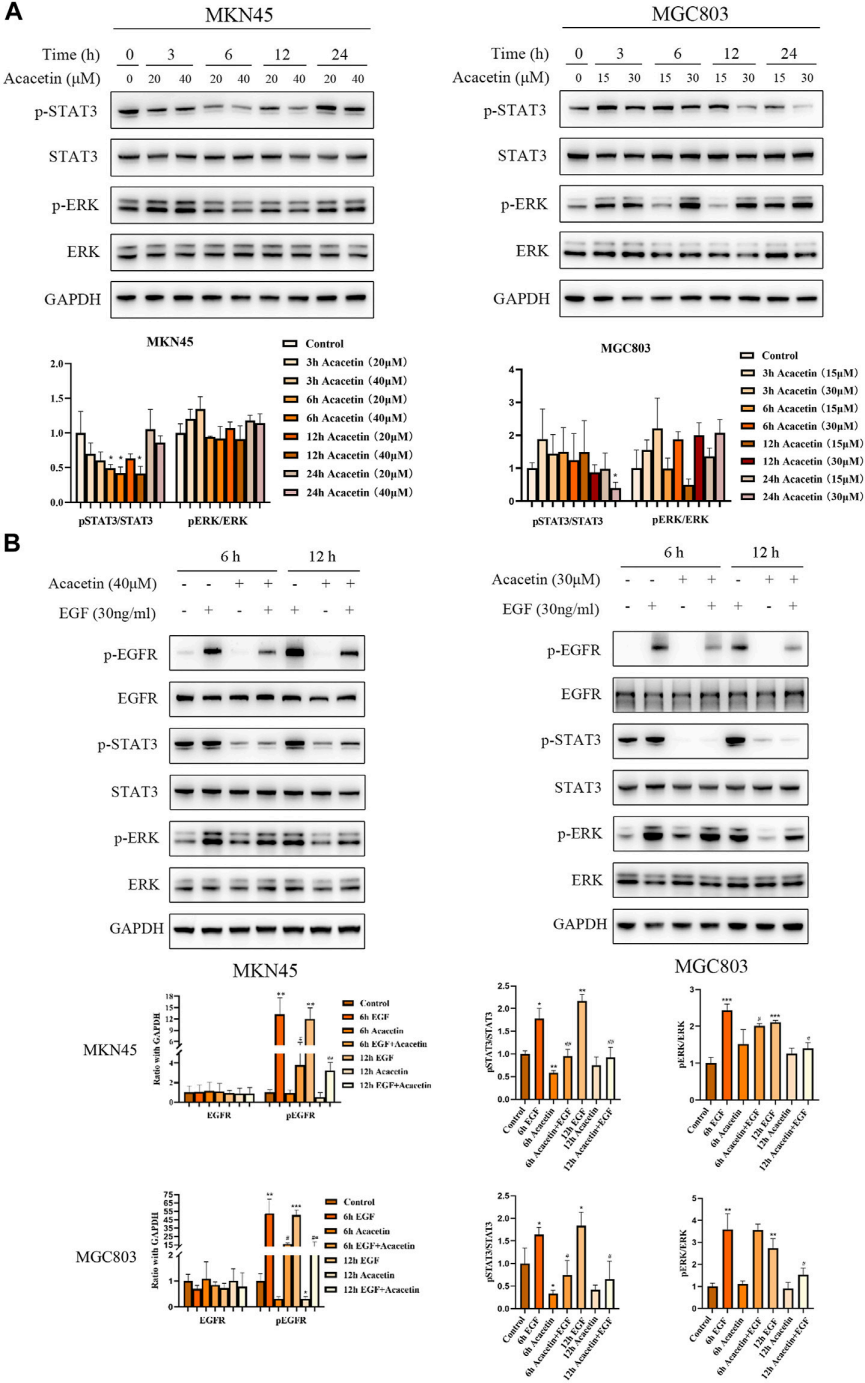
Acacetin played an anticancer role by regulating the STAT3 and ERK pathways. **(A)** Effects of acacetin on the STAT3 and ERK pathways in MKN45 and MGC803 cells after treatment with different concentrations and for different time points. **(B)** Assessment of the effects of acacetin combined with EGF on the levels of phosphorylated STAT3 and ERK pathway-related proteins in GC cells at different time points. *p < 0.05, ^**^ *p* < 0.01 and ^***^ *p* < 0.001 *versus* control. ^#^ *p* < 0.05, ^##^ *p* < 0.01 and ^###^ *p* < 0.001 *versus* the EGF-treated group.

Activated EGFR phosphorylates many crucial signaling molecules, such as JAK2, PI-3K and Ras (Han and Lo, 2012). Our previous studies showed that acacetin inhibits the invasion, migration and TGF-β1-induced EMT of GC cells through the PI3K/Akt/Snail pathway (Zhang et al., 2022). Therefore, this study focused on the effects of acacetin on proteins related to the JAK2 and Ras pathways. Two GC cell lines, namely, the MKN45 and MGC803 cell lines, were treated with different concentrations of acacetin for 3, 6 h, and 12 h. The results showed that the p-STAT3/STAT3 ratio was reduced in a dose- and time-dependent manner in acacetin-treated cells compared with untreated cells (*p* < .05) (Figure 5A). However, the p-ERK/ERK ratio was not significantly decreased in MKN45 cells and tended to increase at some time points in MGC803 cells. Acacetin rapidly and transiently inhibited ERK signaling, and this effect was quickly reversed (Figure 5A). Next, epidermal growth factor (EGF), one of the ligands of EGFR, triggers signal transduction through ERK and STATs and other pathways (Schlessinger, 2000). Therefore, we examined whether acacetin could inhibit EGF-stimulated STAT3 and ERK phosphorylation. We first verified the optimal treatment time and concentration of EGF. According to the experimental results, the optimal treatment time was 5 min and the optimal concentration was 30 ng/mL (Supplementary Figure S5). The results illustrate that EGF stimulation increased the levels of phosphorylated EGFR, STAT3, and ERK, while pretreatment with acacetin significantly inhibited EGF-induced EGFR, STAT3, and ERK activation; however, the inhibition of phosphorylated ERK by acacetin alone was weak (Figure 5B). In conclusion, our results demonstrated that the inhibitory effects of acacetin were likely mediated through the JAK2 and Ras pathways.

### Acacetin inhibits tumor growth *in vivo*

**FIGURE 6:**
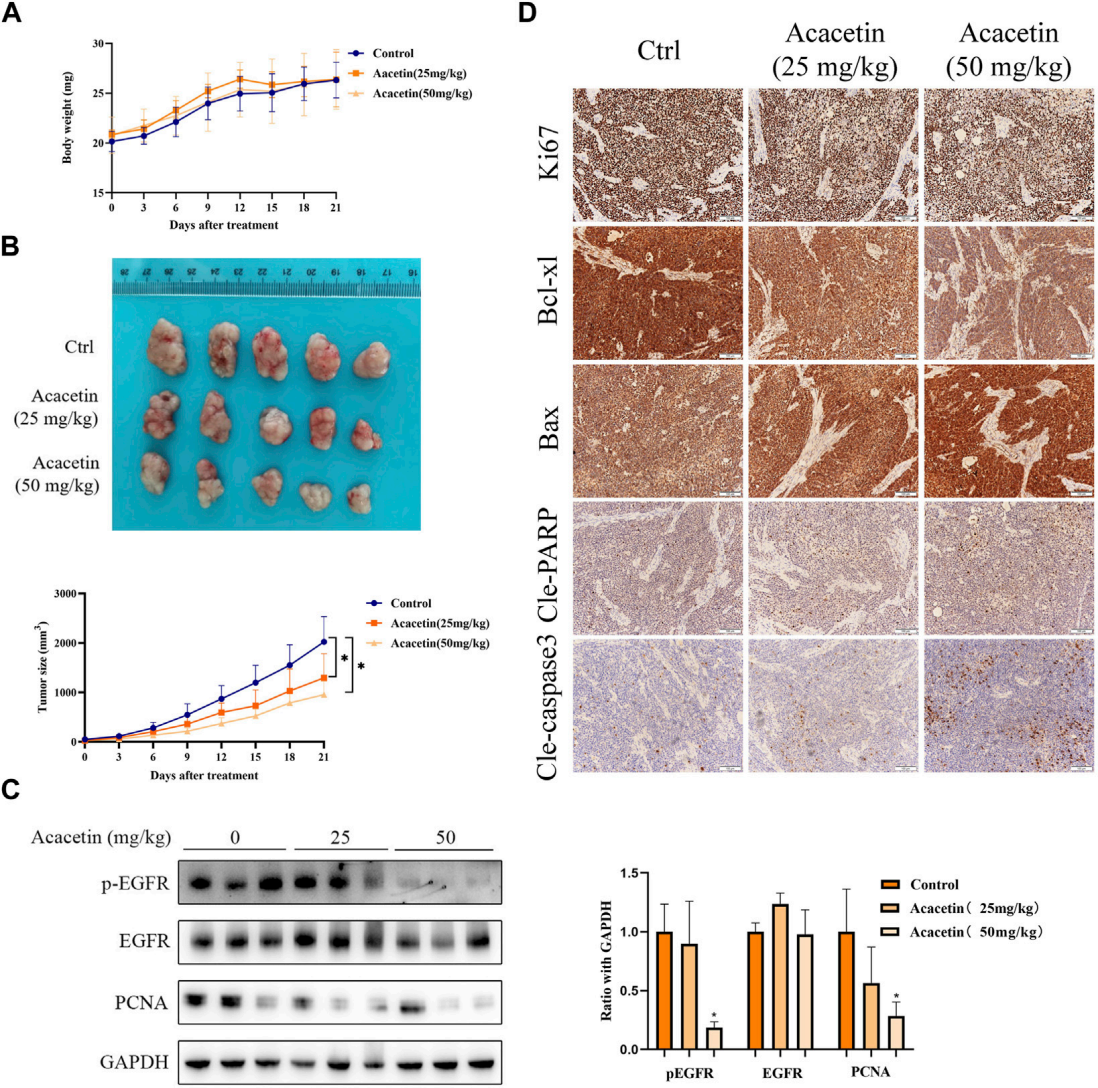
Acacetin suppressed the growth of MKN45 xenograft tumors. **(A)** Changes in the body weight of each group throughout the course of treatment of nude mice. **(B)** Representative images of xenograft tumors after different treatments. Measurement of tumor volume in each group at different time points. **(C)** Western blotting and **(D)** IHC were used to measure the expression of EGFR, pEGFR, and proliferation- and apoptosis-related proteins in each group.

To evaluate the antitumor effect of acacetin *in vivo*, we established a xenograft model by subcutaneously injecting MKN45 cells into BALB/c nude mice. The results indicated that the average tumor size in the 50 mg/kg acacetin group was significantly decreased compared with that in the DMSO control group (*p* < .05). However, the weight of the nude mice in the acacetin group was not affected (Figures 6A, B). In addition, HE staining of the liver, lung and kidney showed no obvious inflammatory cell infiltration, tissue damage or other pathological changes (Supplementary Figure S6). Serum levels of alanine aminotransferase (ALT) and aspartate aminotransferase (AST) showed an increasing trend after treatment with 50 mg/kg acacetin, but the absolute value did not exceed 1 ng/mL (Supplementary Figure S7). Then, Western blotting and IHC staining were carried out to further assess the mechanism by which acacetin inhibits xenograft tumors. We randomly examined three tumors in each group. The Western blotting results showed that the expression of p-EGFR and PCNA (proliferating cell nuclear antigen) in the subcutaneous tumor tissues was significantly decreased in the acacetin group (50 mg/kg) compared with the control group (*p* < 0.05; Figure 6C). The IHC analysis revealed that acacetin significantly decreased the expression of Bcl-xl and increased the expression of Bax, cleaved PARP and cleaved caspase3, which was consistent with the *in vitro* data. The Ki67 proliferation index decreased from more than 90% (control group) to approximately 60% (50 mg/kg acacetin group; Figure 6D). Taken together, these data proved that acacetin suppressed GC xenograft tumor growth by decreasing EGFR phosphorylation *in vivo*.

## Discussion

**FIGURE 7:**
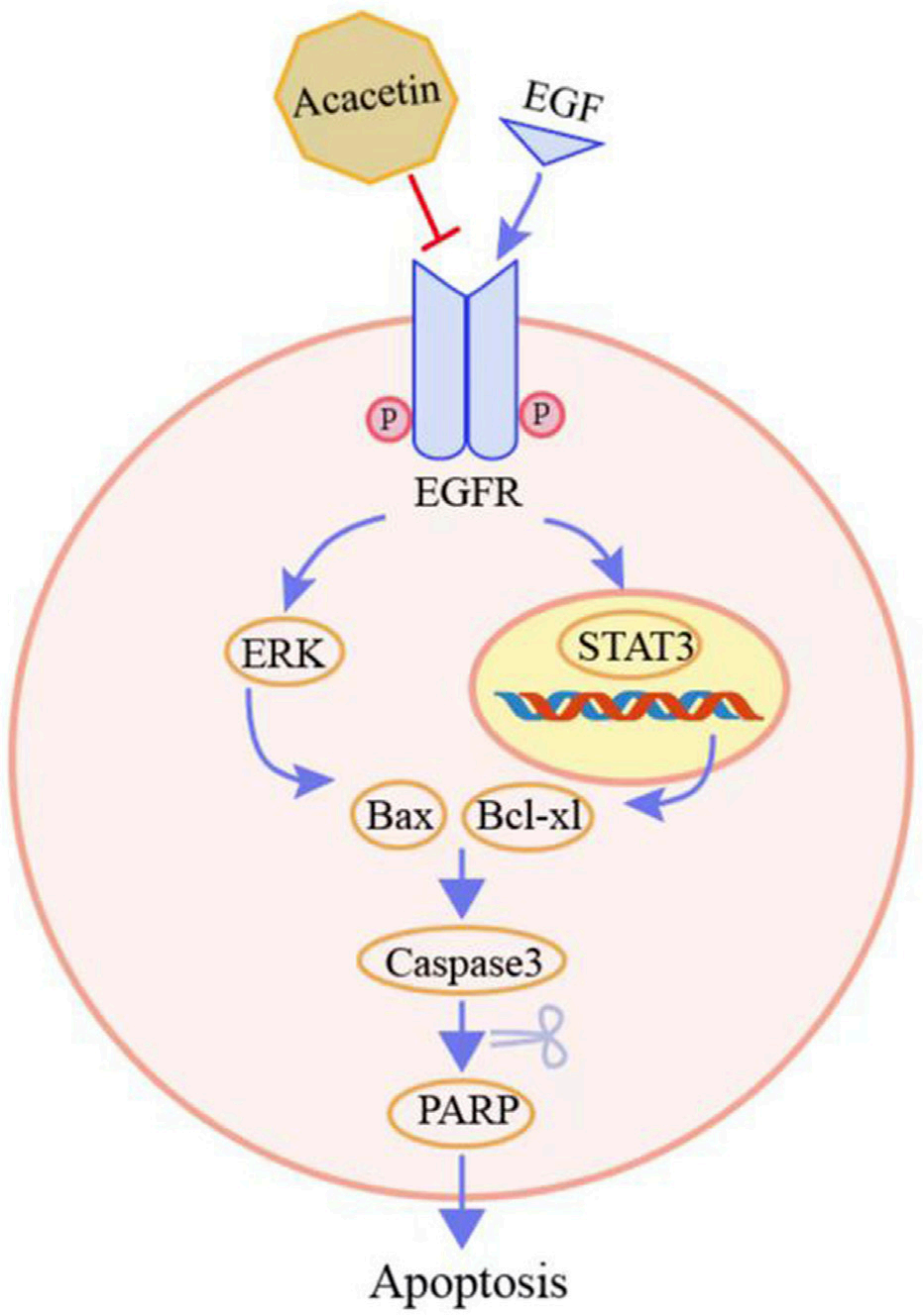
The process of exploring and validating the targets of acacetin.

Accumulating evidence suggests that acacetin exerts antitumor effects (Alfwuaires et al., 2022). However, the inhibitory effects of acacetin on GC, and the specific molecular mechanisms, remain largely unknown. Natural products often have multiple molecular targets. A previous study found that acacetin functions as a STAT3 inhibitor by directly binding to the STAT3 protein in prostate cancer cells (Yun et al., 2021). Therefore, in this study, the effective targets of acacetin were identified by combining target prediction and experimental verification. We first confirmed that acacetin has two other targets, EGFR and Met. The mechanism underlying the antitumor effects of acacetin binding to EGFR was proven by cell and animal experiments (Figure 7).

The identification of protein targets of natural products is challenging, but it is essential for understanding their functions. The network pharmacology approach has been successfully used in the search for the therapeutic targets of bioactive ingredients according to the “disease-target-ingredient drug” network model (Hopkins, 2008). In addition, we hypothesized that the proteins that are encoded by differentially expressed genes, according to RNA sequencing, might be the direct targets of acacetin. Therefore, targets derived from network pharmacology and RNA sequencing were selected for subsequent experiments.

Identification of the target proteins of label-free natural products is performed by methods that include DARTS, CETSA, stability of proteins from rates of oxidation (SPROX), etc. (Chang et al., 2016). Among these approaches, the principle of DARTS is based on the fact that bound target proteins exhibit reduced susceptibility to proteases compared to unbound proteins (Lomenick et al., 2009). Despite its limitations in identifying low-abundance proteins, DARTS is still a reliable method for the determination of the target proteins of natural products (Chin et al., 2014). In this project, the predicted targets of acacetin were screened by DARTS, and two targets, EGFR and Met, were ultimately identified. Subsequent experiments proved that acacetin had a great effect on EGFR, so we selected this target for further study. Another method, CETSA, which is based on the ligand-induced thermodynamic stabilization of target proteins (Martinez Molina et al., 2013), also demonstrated that acacetin binds to EGFR. In addition, the binding activity between acacetin and EGFR was verified to be good by molecular docking.

EGFR, which is associated with cell proliferation and survival, is overexpressed in many epithelial tumors (Weihua et al., 2008). Therefore, EGFR is the most well-studied tyrosine kinase receptor whose signaling is involved in neoplasia (Levantini et al., 2022). EGFR has been reported to be overexpressed in 3%–5% of GC cases, and EGFR overexpression is correlated with poor prognosis (Chen et al., 2013; Schrock et al., 2017). EGFR-targeted therapies are a developing area of research, and these therapies have the potential to be utilized in the treatment of GC. Our findings demonstrated that acacetin not only binds to EGFR but also inhibits its phosphorylation in GC. In the reverse experiment, EGF-activated EGFR phosphorylation was also suppressed.

Inhibition of EGFR can lead to proliferation arrest and apoptosis (Arteaga and Engelman, 2014). Therefore, this study focused on the effect of acacetin on GC cell apoptosis. *In vitro* experiments confirmed that acacetin could inhibit the growth of GC cells both in the short and long term. These results are related to the regulation of Caspase and PARP signaling by acacetin. The activation of multiple signaling pathways downstream of EGFR is closely related to apoptosis (Al Zaid Siddiquee and Turkson, 2008; Lavoie et al., 2020). Then, we found that acacetin inhibited STAT3 phosphorylation. Although the inhibition of the ERK pathway by acacetin was transient and quickly reversed, it is required for the rapid induction of apoptosis. Combined with reverse experiments, these results demonstrated that acacetin could promote apoptosis through the ERK and STAT3 pathways (Will et al., 2014).

Notably, acacetin showed satisfactory therapeutic results *in vivo* without causing clear organ toxicity at the therapeutic concentration used in this study. Although the ALT and AST levels were slightly increased in the acacetin treatment group (50 mg/kg), the absolute values were still lower than 1 ng/mL, and there was no obvious pathological damage in the liver. Western blotting and immunohistochemical analyses proved that acacetin could inhibit proliferation and promote apoptosis. Combined with our previous study, these findings provide strong evidence that acacetin has the potential to exert therapeutic effects on GC.

## Conclusion

In summary, through target prediction and experimental verification, we identified a direct target of acacetin in gastric cancer cells. Acacetin significantly promoted GC cell apoptosis by regulating the STAT3 and ERK signaling pathways. These findings highlight the important potential of acacetin to be used as a drug candidate for the treatment of GC.

## Data Availability

The datasets presented in this study can be found in online repositories. The names of the repository/repositories and accession number(s) can be found below: https://www.ncbi.nlm.nih.gov/bioproject/PRJNA912885.

## Abbreviations

GC, Gastric cancer; DARTS, drug affinity responsive target stability; CETSA, cellular thermal shift assay; ELISA, Enzyme-linked immunosorbent assay; PPI, protein‒protein interaction; GO, Gene Ontology; KEGG, Kyoto Encyclopedia of Genes and Genomes; EGF, epidermal growth factor; STAT3, Signal transducer and activator of transcription 3; EMT: epithelial-to-mesenchymal transition; TGF-β1, transforming growth factor-β1; CCK-8, Cell Counting Kit-8; MMP, matrix metalloproteinase; DAPI, 4′,6‐diamidino‐2‐phenylindole; IC50, half inhibitory concentration; ALT, alanine aminotransferase; AST, aspartate aminotransferase.
