# Supplementary material for: Acacetin exerts antitumor effects on gastric cancer by targeting EGFR
Source: Front Pharmacol. 2023 May 17;14:1121643. doi: 10.3389/fphar.2023.1121643 (PMC10231641; doi:10.3389/fphar.2023.1121643)
Supplement: Supplementary file 1 [file DataSheet1.docx]

**Supplement materials**


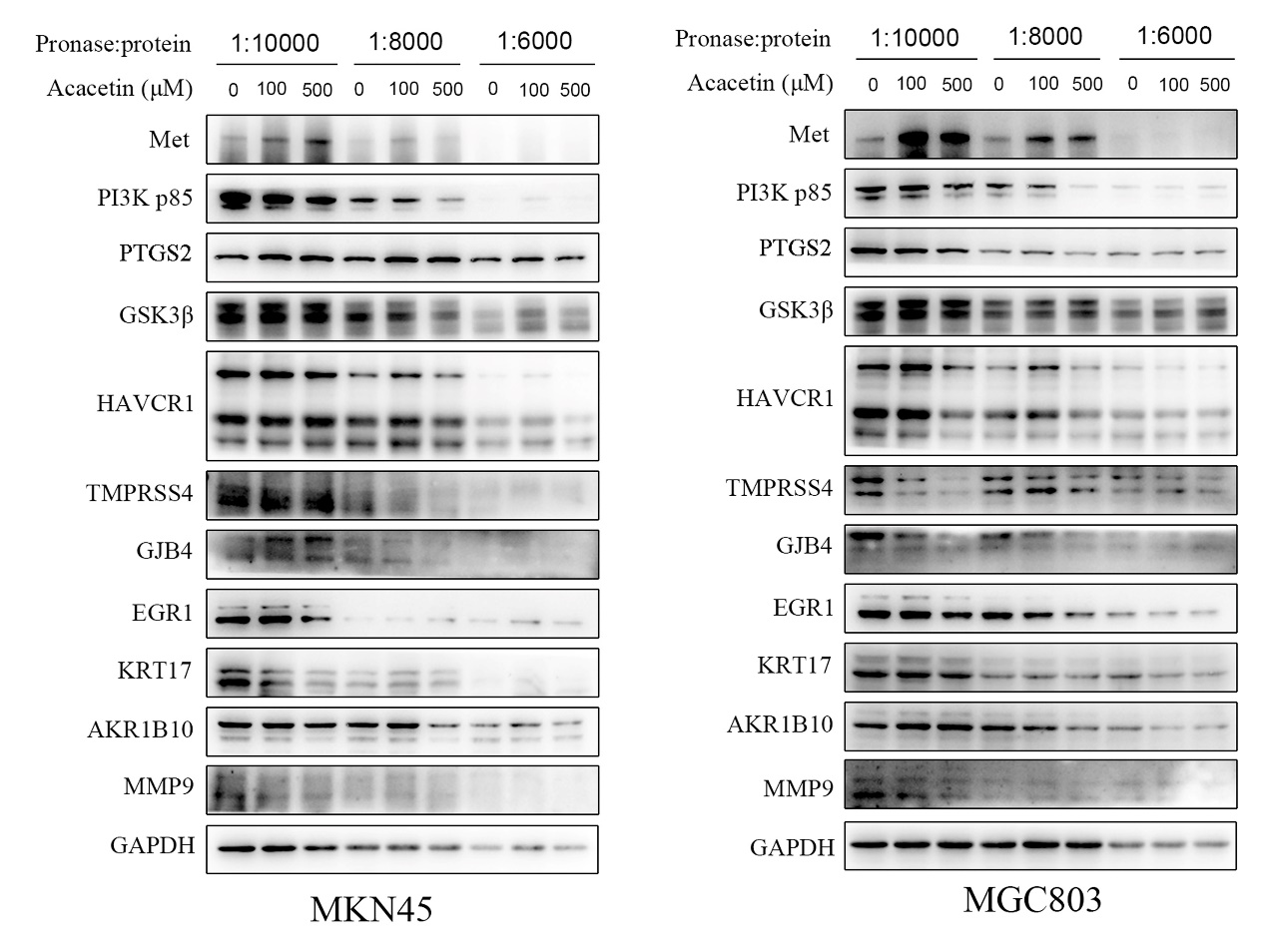
Figure S1. DARTS assay to verify the network pharmacology and RNA sequencing derived drug targets of acacetin.


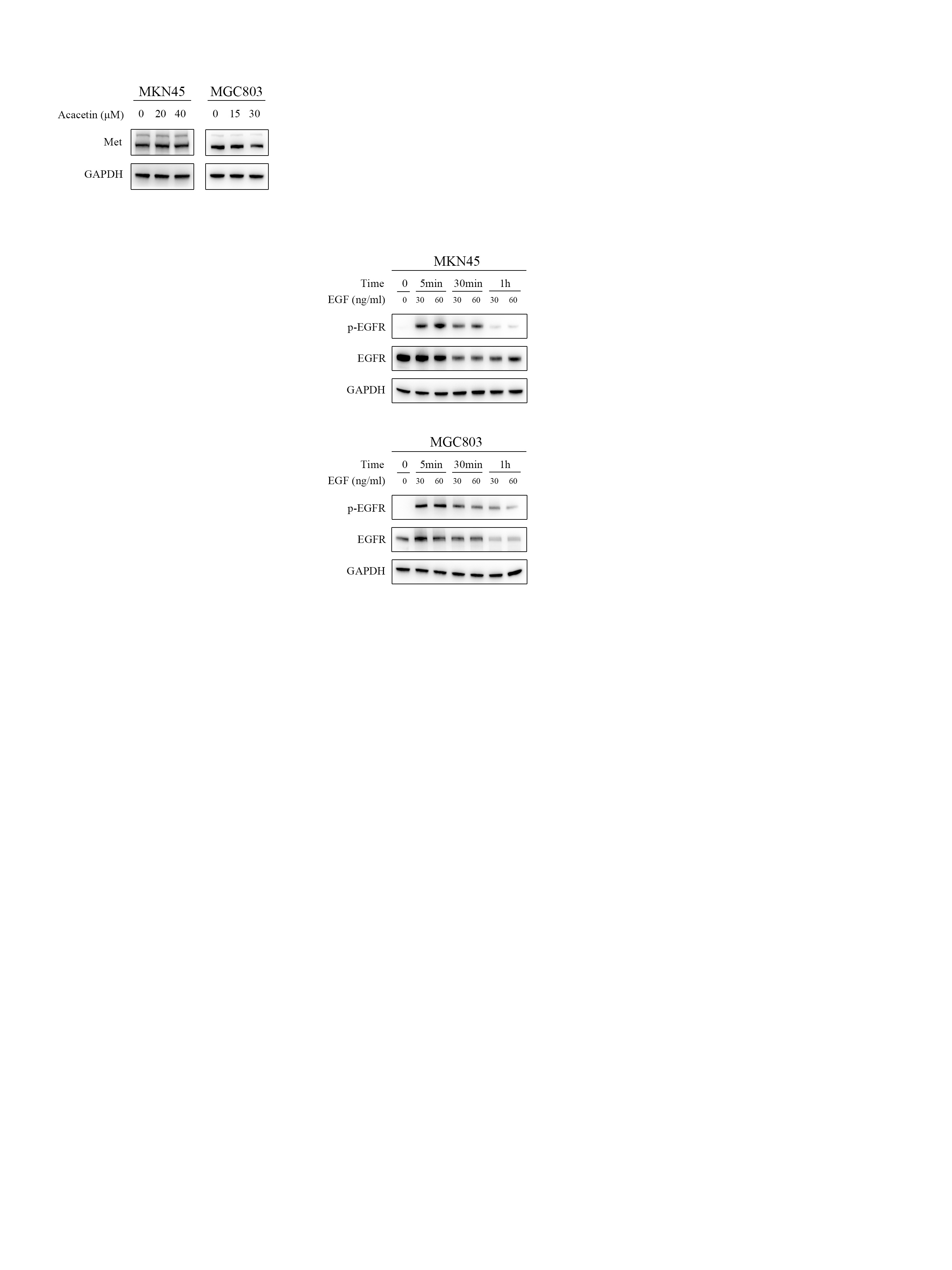


Figure S2. Evaluation of the effects of acacetin on the Met protein by western blotting.


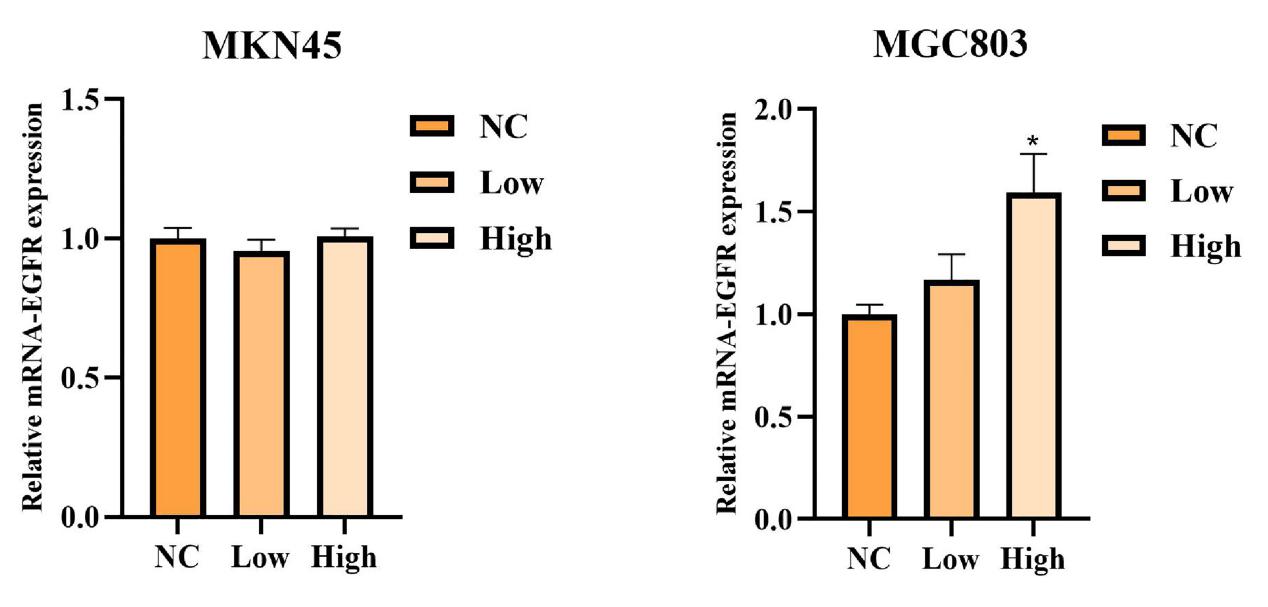


Figure S3. The expression of EGFR in MKN45 and MGC803 cells after acacetin intervention.


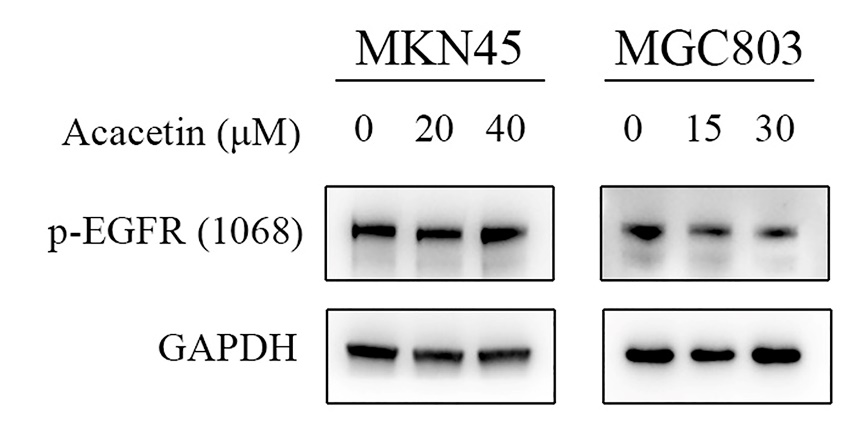


Figure S4. Assessment of the effect of acacetin on residue 1068 of EGFR.


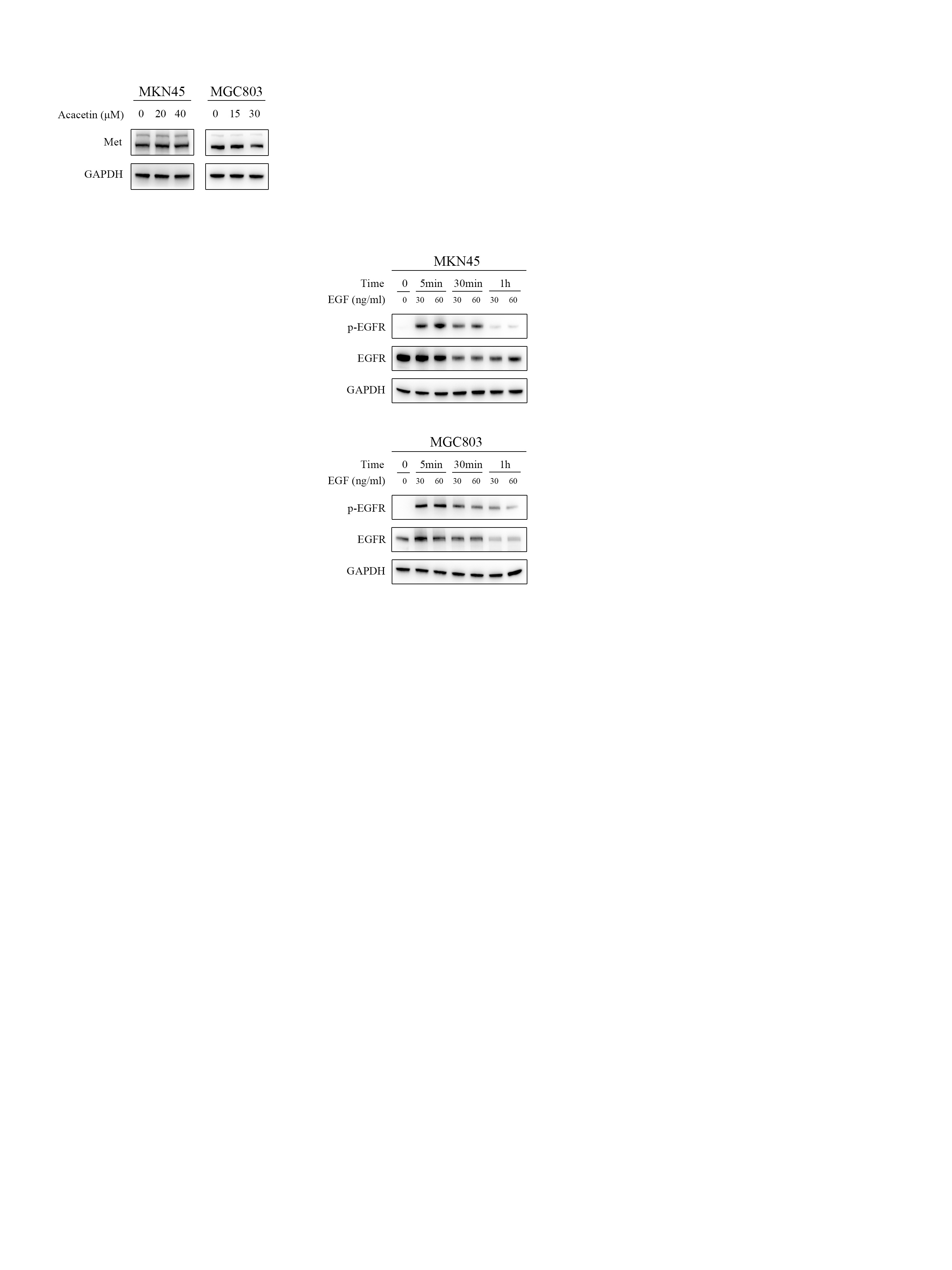

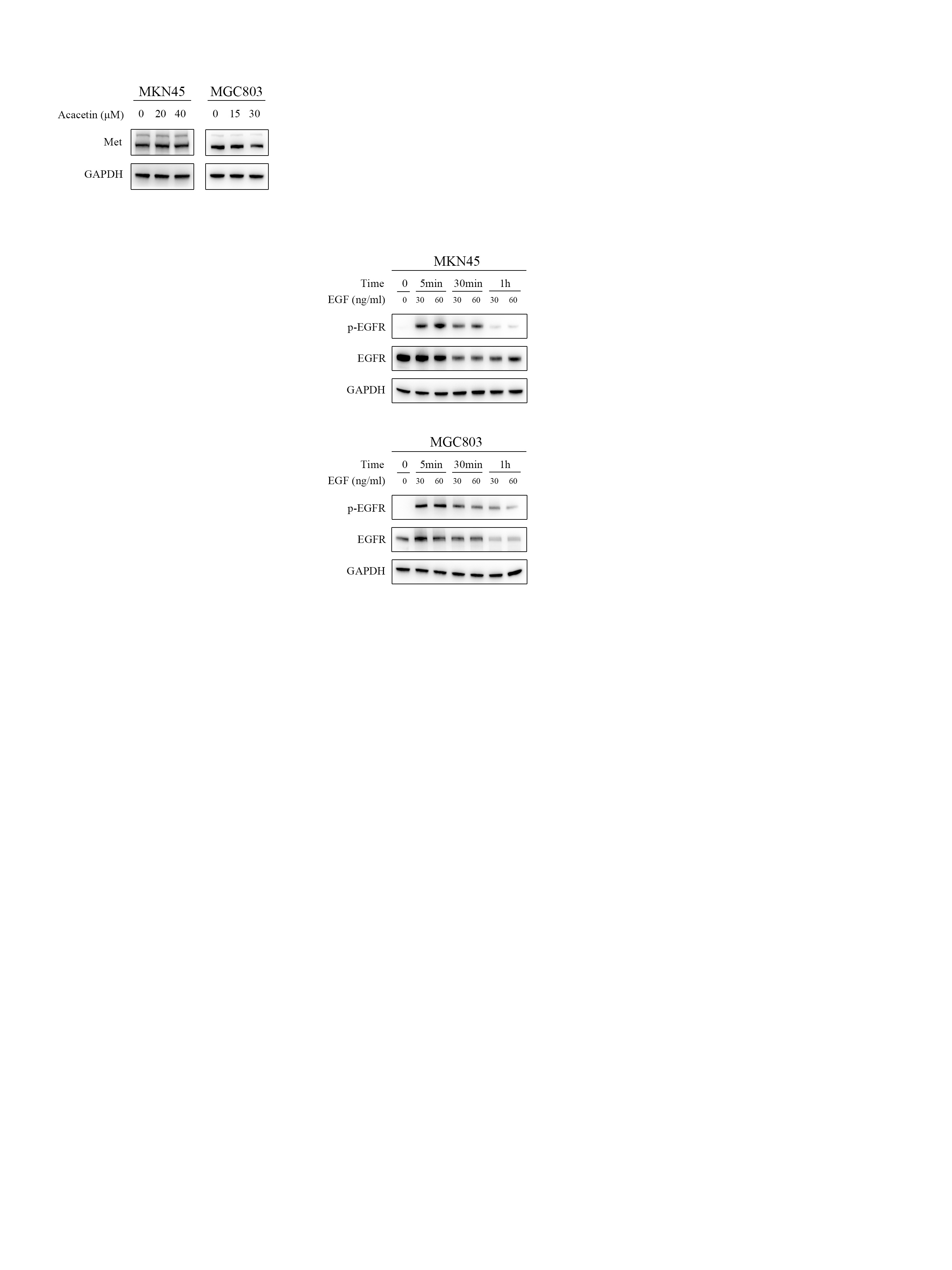


Figure S5. Effects of EGF on GC cells after treatment with different concentrations and for different time points.


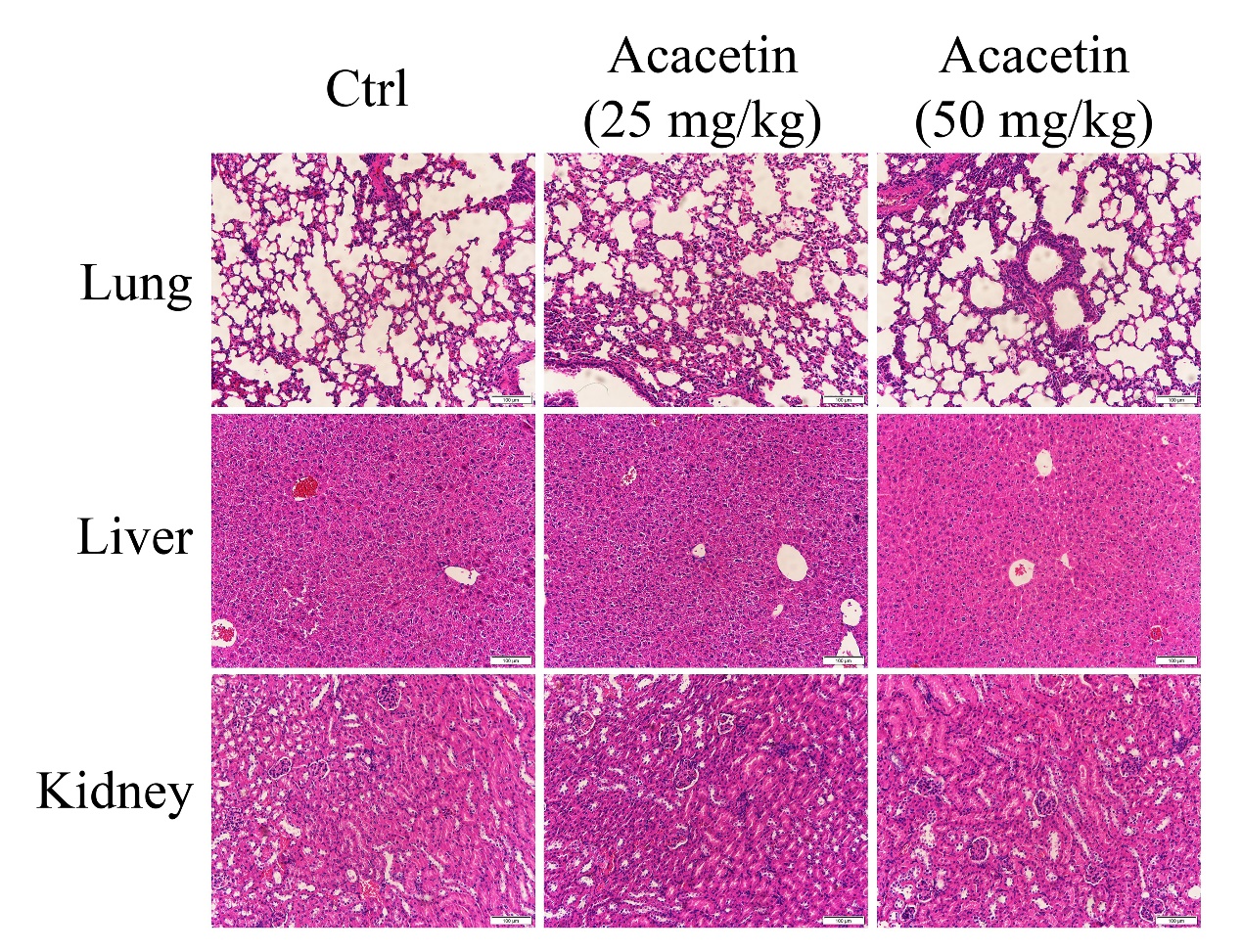


Figure S6. HE staining of the liver, lung and kidney.


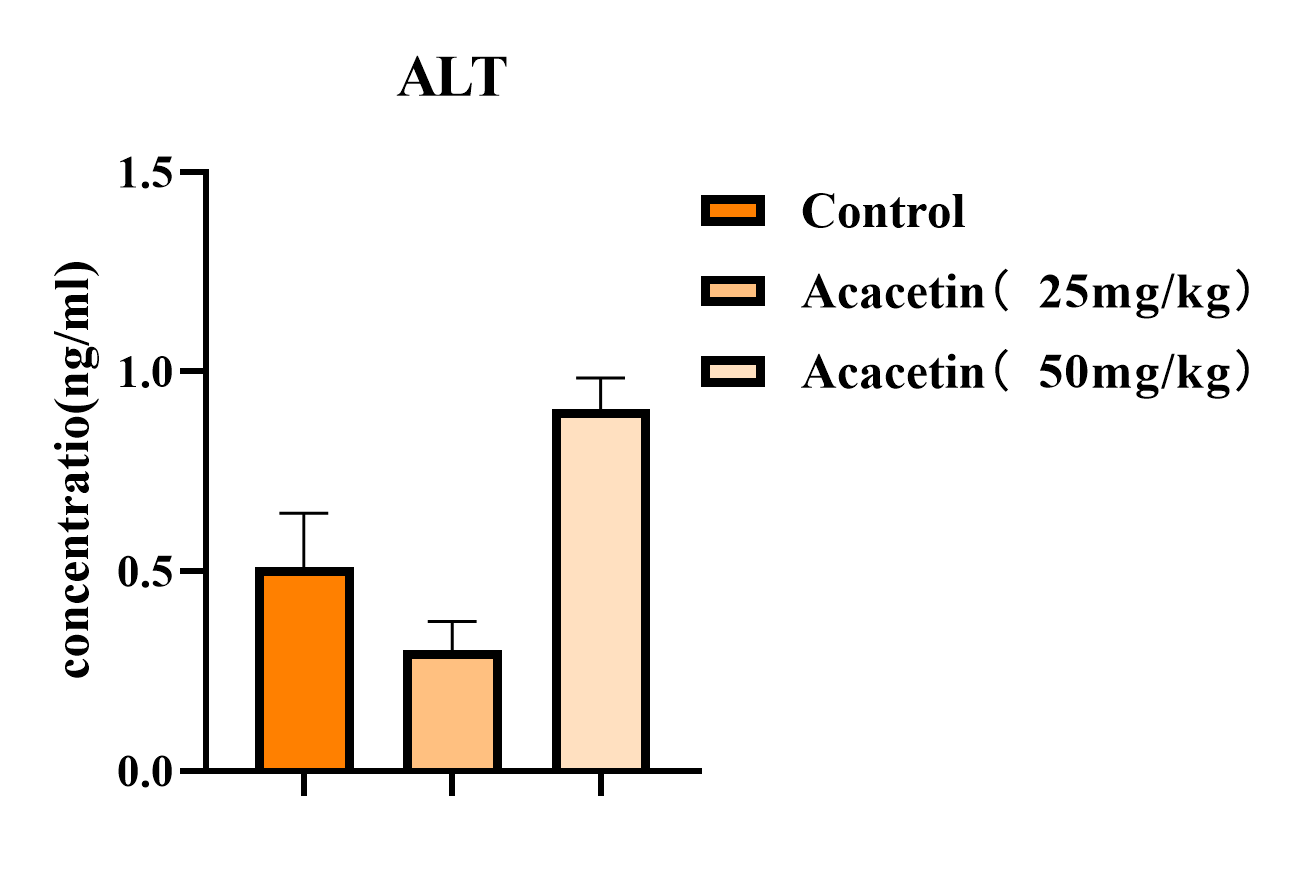

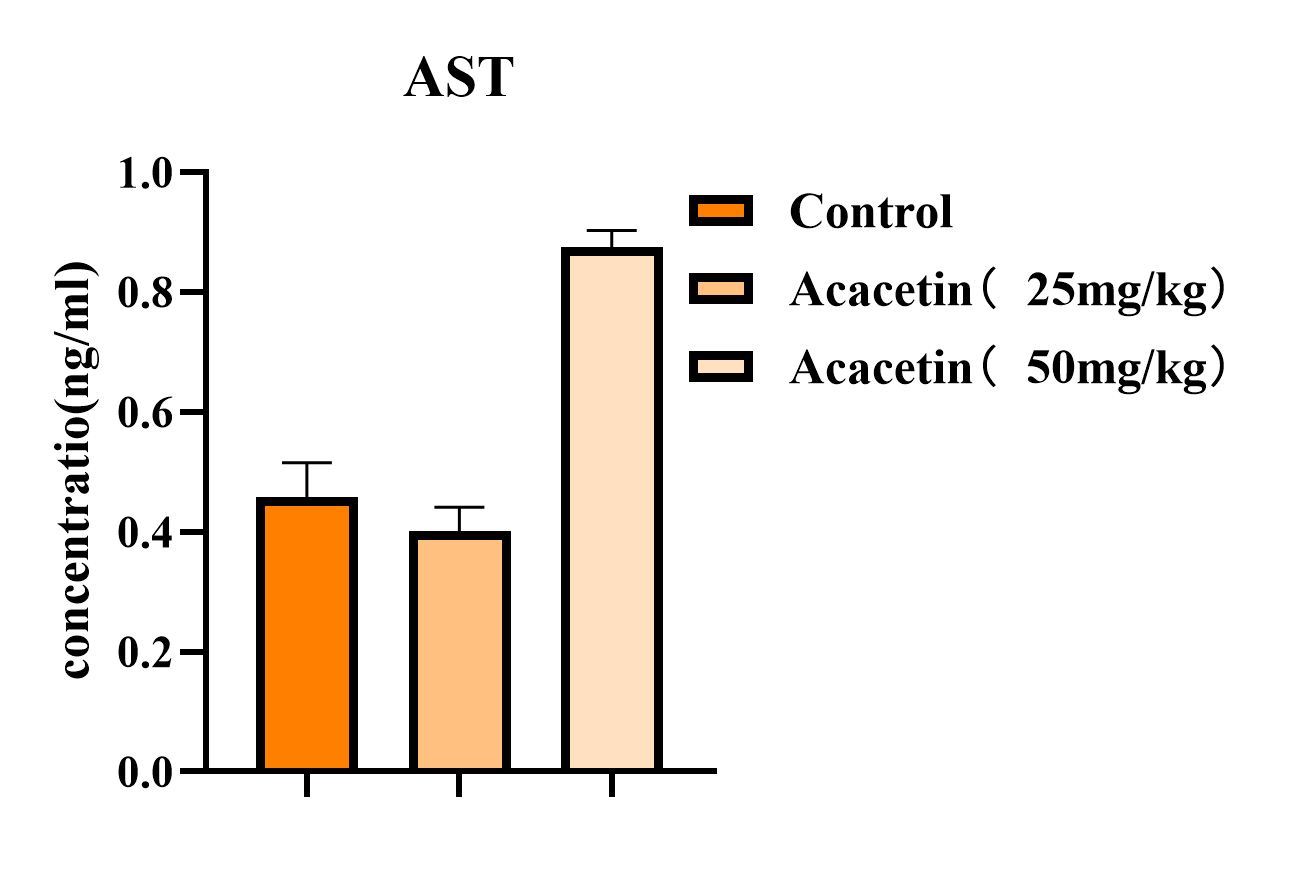


Figure S7. The serum contents of ALT and AST were measured by ELISA.
